# Various Strategies for the Immobilization of a Phospholipase C from *Bacillus cereus* for the Modulation of Its Biochemical Properties

**DOI:** 10.3390/molecules29071467

**Published:** 2024-03-26

**Authors:** Ines Abdelkader, Jose M. Guisán, Adel Sayari, Gloria Fernández-Lorente

**Affiliations:** 1 Laboratory of Biochemistry and Enzymatic Engineering of Lipases, National School of Engineers of Sfax, University of Sfax, PB 1173, Km 4 Road Soukra, Sfax 3038, Tunisia; inesabdelkader1@gmail.com (I.A.); adelsayari3@gmail.com (A.S.); 2 Department of Biocatalysis, Institute of Catalysis and Petrochemistry (ICP, CSIC), Marie Curie, 2, UAM Campus, Cantoblanco, 28049 Madrid, Spain; jmguisan@icp.csic.es; 3 Laboratory of Microbiology and Food Biocatalysis, Institute of Food Science Research (CIAL, CSIC-UAM), Nicolás Cabrera, 9, UAM Campus, Cantoblanco, 28049 Madrid, Spain

**Keywords:** phospholipase C, immobilization, stabilization, biochemical characterization

## Abstract

In this study, the effect of various immobilization methods on the biochemical properties of phospholipase C (PLC) from *Bacillus cereus* obtained from the oily soil located in Sfax, Tunisia, was described. Different supports were checked: octyl sepharose, glyoxyl agarose in the presence of N-acetyl cysteine, and Q-sepharose. In the immobilization by hydrophobic adsorption, a hyperactivation of the PLC_Bc_ was obtained with a fold of around 2 times. The recovery activity after immobilization on Q-sepharose and glyoxyl agarose in the presence of N-acetyl cysteine was 80% and 58%, respectively. Furthermore, the biochemical characterization showed an important improvement in the three immobilized enzymes. The performance of the various immobilized PLC_Bc_ was compared with the soluble enzyme. The derivatives acquired using Q-sepharose, octyl sepharose, and glyoxyl agarose were stable at 50 °C, 60 °C, and 70 °C. Nevertheless, the three derivatives were more stable in a large range of pH than the soluble enzyme. The three derivatives and the free enzyme were stable in 50% (*v*/*v*) ethanol, hexane, methanol, and acetone. The glyoxyl agarose derivative showed high long-term storage at 4 °C, with an activity of 60% after 19 days. These results suggest the sustainable biotechnological application of the developed immobilized enzyme.

## 1. Introduction 

Enzymes have excellent catalytic properties such as activity, selectivity, and specificity [1] that may allow them to catalyze compound chemical reactions. However, most of the enzymes are inappropriate for industrial applications for some reason; they are soluble and frequently unstable in harsh conditions, like high temperatures or the presence of organic solvents [2,3]. Nevertheless, this problem could be solved by immobilization. In fact, the immobilization of enzymes has appeared as an alternative to augment their biochemical properties and also their operational activity [4]. Phospholipases are hydrolase enzymes that catalyze phospholipids. Depending on the bond, it is cleaved into four major families (A, B, C, and D) [5]. Phospholipase Cs (PLCs) catalyze the hydrolysis of the glycerophosphate bonds of phospholipids to liberate phosphomonoester and diacylglycerol [6]. During the past decades, PLCs have been increasing due to their potential applications as multipurpose biocatalysts in several industrial fields, such as nutraceuticals, food additives, ceramide production, and oil degumming [5]. Nowadays, immobilization is a widespread technology for enzymes. It could be defined as the process of attaching or confining an enzyme to a solid support or matrix. This approach is considered an efficient method because it enhances operational characteristics such as stability and storage; it is also easy to manipulate and avoid aggregation [7,8]. In addition, the procedure of enzyme immobilization can improve enzyme activity; it also offers the possibility of reusing the support and the enzyme on several cycles through recovering the enzyme from the reaction mixture [9,10].

**Figure 1:**
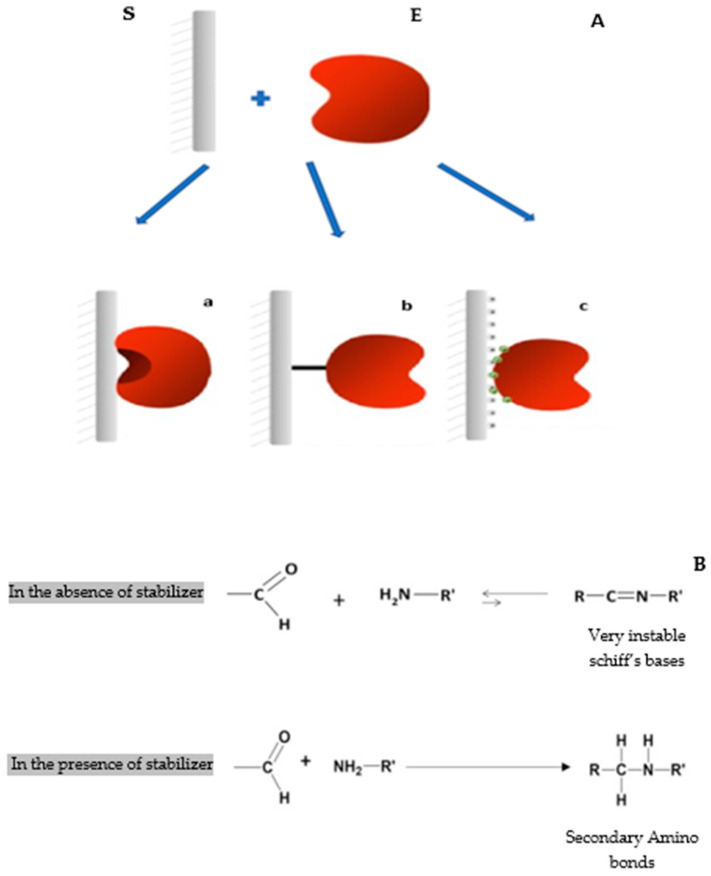
(**A**) Various immobilization techniques used in this study. S: support; E: enzyme. (**a**) Immobilization via interfacial activation on hydrophobic supports on octyl sepharose; (**b**) immobilization by covalent attachment on glyoxyl agarose; (**c**) immobilization via ionic exchange on Q-sepharose. (**B**) The effect of absence and presence of a reducing agent for the formation of secondary amino bonds during immobilization by covalent attachment.

Several reversible and irreversible immobilization approaches have been involved in enzyme immobilization [11,12,13,14]. One of the most commonly used techniques in the immobilization of lipases and some phospholipases is their immobilization on hydrophobic supports via interfacial activation. This immobilization process has several advantages [13]; in most cases, it facilitates the enzyme’s purification [13,15]. This kind of immobilization can cause stabilization, hyperactivation, and also the modulation of the biochemical proprieties of the enzyme through its fixation on the open form of the support [9] (Figure 1(Aa)). The technique of immobilization using glyoxyl agarose as a support is considered an excellent technique to stabilize enzymes [16]. Thus, in some cases, glyoxyl agarose derivatives can be more stable than the soluble enzyme under harsh conditions such as high temperatures or the presence of an extreme concentration of organic solvents (>50% *v*/*v*) [13,17]. Nevertheless, immobilization on glyoxyl agarose has a critical disadvantage, for the reason that it cannot be carried out at a neutral pH when most of the enzymes are stable [18]. Such a restriction depends on the reversible nature of the imine bond between the enzyme and the support. Also, the primary amine (nucleophilic groups) of the enzyme has a low reactivity at pH 7 against the aldehyde groups of the glyoxyl-agarose support [16]. For this reason, the immobilization of enzymes with the littlest physicochemical modifications on glyoxyl agarose supports is suggested. One-point covalent immobilization of enzymes on aldehyde-agarose supports (at slightly alkaline pH 8.5) is here proposed as a suitable protocol to prepare immobilized “native enzymes” (Figure 1(Ab)). After immobilization, derivatives are reduced with sodium borohydride. In this way, the primary amino terminus of the native enzyme is simply converted into a secondary amino group (with very similar physical properties), and the enzyme surface remains unaltered in the proximity of a fully inert and highly hydrophilic internal surface of agarose gels. One-point attachment between one amino group and one linear aldehyde group yields a very unstable Schiff’s base. However, these single Schiff’s bases can be stabilized in order to promote stable one-point enzyme-agarose immobilization by the formation of stable complexes between Schiff’s bases and thiolated compounds such as acetylcysteine, mercaptoethanol, or dithiothreitol (DTT) [19]. The immobilized derivatives have to be reduced with sodium borohydride after the one-point covalent immobilization in order to reduce Schiff’s bases to secondary amino groups and to lower the remaining aldehyde groups into hydroxyl ones (Figure 1B) [19].

Ionic exchange chromatography matrices could be considered a gentle method for immobilizing enzymes. This technique of immobilization involves the interaction between the charged groups on the enzyme and the charged group on the ion exchange resin. In fact, immobilization of enzymes on ion exchange chromatography provides a reversible and selective means of immobilization, allowing the purification and separation of the enzyme. In addition, ionic exchange immobilization represents a rapid and sample method for protein separation that is highly activated with very strong ionic groups; therefore, it can adsorb the maximum percentage of proteins from the extract [20] (Figure 1(Ac)). The most common ion exchange chromatographies utilized for enzyme immobilization and purification are Q-sepharose, DEAE-cellulose, and CM-cellulose.

On the other hand, enzymes as industrial biocatalysts offer numerous advantages over traditional chemical processes with respect to sustainability and process efficiency. Enzyme catalysis has been scaled up for commercial processes; among the enzymes with industrial interest, PLCs enjoy a privileged position. Microbial PLCs have gained considerable potential for industrial applications such as degumming in the processing of food, vegetable oil, and pharmaceutical sciences [21,22]. Oil degumming is the first step in the vegetable oil refining process, which necessitates the elimination of phospholipids and mucilaginous gums [23,24]. Because the presence of phospholipids in a high quantity in the final product can cause oil discoloration and adversely affect its quality [22,24].

Enzymatic degumming using PLCs has many benefits. It presents an efficient and eco-friendly process for vegetable oil refining [25]. For example, in crude soybean oil, which contains a high quantity of phospholipids, the hydrolysis of those phospholipids with the PLCs produces DAGs that are mixable with TAGs that could be recovered and increase the oil yield [26]. PLCs from *Bacillus cereus* are considered the most common enzymes used for the degumming process of vegetable oils [27] due to their high activity, substrate spectrum, and approved safety [27]. 

In this work, a novel phospholipase C from *Bacillus cereus* was produced as described previously [28]. Then, we immobilized the enzyme using different methods of immobilization (Figure 1). In this way, the effect of various immobilization methods on the biochemical characteristics of PLC_Bc_ was evaluated. Biocatalytic properties included stability against pH, thermostability, the presence of high concentrations of solvents, and enzyme storage. In fact, the different derivatives were compared to the soluble enzyme. The objective of this work is to explore the future possibilities of the immobilized enzyme in biotechnology applications. 

## 2. Results

### 2.1. Immobilization of PLC_Bc_ on Different Supports

**Figure 2:**
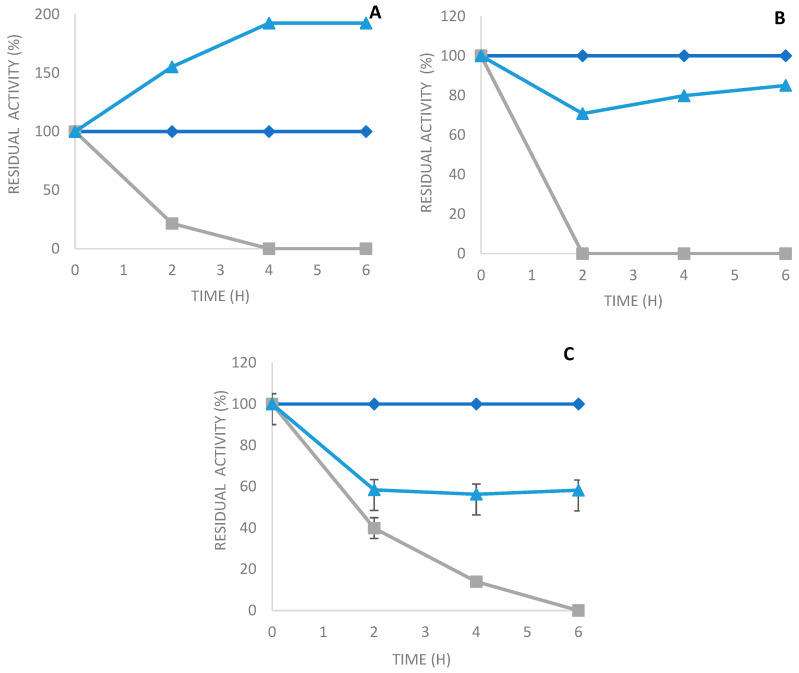
Immobilization kinetics of PLC_Bc_ on (**A**) octyl sepharose; (**B**) Q-sepharose; and (**C**) glyoxyl agarose in the presence of N-acetyl cysteine. Gray: supernatant, blue triangles: suspension, and blue squares: soluble enzyme.

The phospholipase C from Bacillus cereus was produced under optimized conditions, as described in a previous study [28]. The crude phospholipase C extract was obtained after centrifugation, as explained in Section 3.2. Later, the extracellular PLC_Bc_ was precipitated by adding ammonium sulfate with a saturation of 80%, followed by dialysis at 4 °C. Then, the PLC_Bc_ was immobilized on different supports using various methods. The immobilization course of PLC_Bc_ on various supports was studied; Figure 2 shows the immobilization kinetics of PLC_Bc_ on octyl sepharose (A), Q-sepharose (B), and glyoxyl agarose in the presence of N-acetyl cysteine (C). The recovery activity after the immobilization of PLC_Bc_ on an octyl sepharose support reached a ratio of approximately 192.5% after 4 h of the immobilization process (Table 1). The activity of PLC_Bc_ immobilized on the octyl sepharose support was around 2-fold higher than the activity of the free enzyme. These results are in good accordance with previous studies using immobilization on octyl sepharose supports [4,29]. Reversible immobilization through the ionic interaction of PLC_Bc_ on Q-sepharose totaled around 80% of the recovery activity obtained after 2 h (Table 1). The covalent immobilization of PLC_Bc_ on glyoxyl agarose in the presence of N-acetyl cysteine produced a slight decrease in recovery activity when compared to the two just-cited supports. 

The decrease could be explained by covalent attachment. In this strategy, the lysine group of the enzyme surface reacts covalently with the glyoxyl group of the support through several attachments. And it can cause a distortion of the structure of the enzyme, related to a slight loss of activity. The hyperactivation of enzymes exhibited after immobilization on hydrophobic supports was found in many studies; for example, the immobilization using hydrophobic supports of some enzymes permits a higher activity of the immobilized enzymes [30,31,32]. As a matter of fact, these supports require the adsorption of the hydrophobic areas, permitting the stabilization of the lid-opened form of the enzyme and exposing the active center of the enzyme to the reactional medium. 

**Figure 3:**
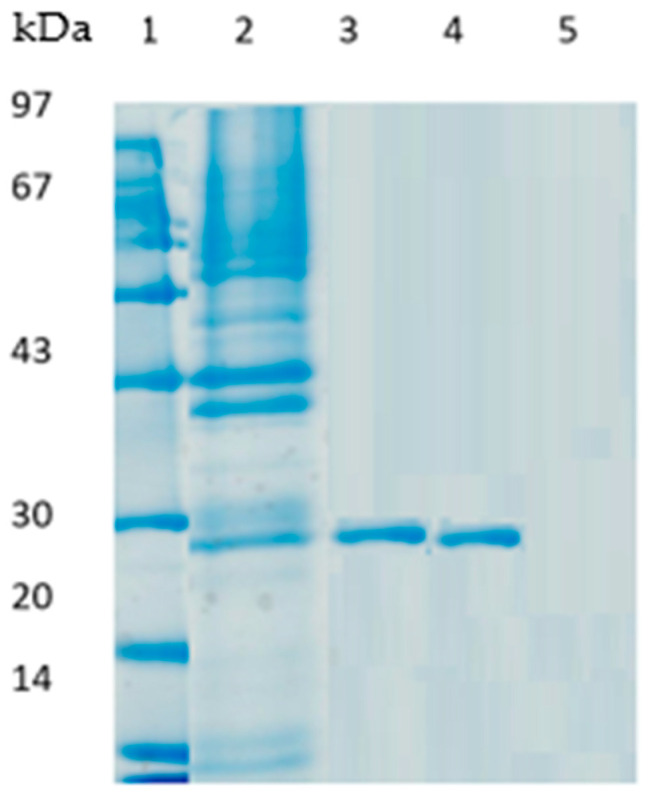
SDS-PAGE analysis of PLC_Bc_ soluble and immobilized on different supports. Lane 1: protein molecular mass marker. Lane 2: free PLC_Bc_ extract; Lane 3: PLC_Bc_ immobilized on octyl sepharose, Lane 4: PLC_Bc_ immobilized on Q-sepharose, Lane 5: PLC_Bc_ immobilized on glyoxyl agarose in the presence of N-acetyl cysteine.

Figure 3 represents an electrophoresis gel of the PLC_Bc_ initial extract and the various derivates of PLC_Bc_. PLC_Bc_ extract immobilized both on octyl sepharose and Q sepharose revealed the presence of only one protein band, with a molecular weight of around 27 kDa (lanes 3 and 4, respectively). In the case of immobilization by hydrophobic adsorption, it has been described that this method is used as a purification method [13]. Because the immobilization process is of low ionic strength, the proteins do not have any exposed hydrophobic regions to be absorbed. While the lipase does, the area close to the lid achieves selective immobilization and thus purification.

Furthermore, the SDS-PAGE profile of PLC_Bc_ immobilized on glyoxyl agarose in the presence of N-acetylcysteine shows the absence of protein bands (lane 5). These results have been proven by other authors [29,33], and they can be explained by the irreversible fixation of the enzyme on the support

### 2.2. Effect of pH on the Activity of PLC_Bc_ Immobilized on Different Supports

**Figure 4:**
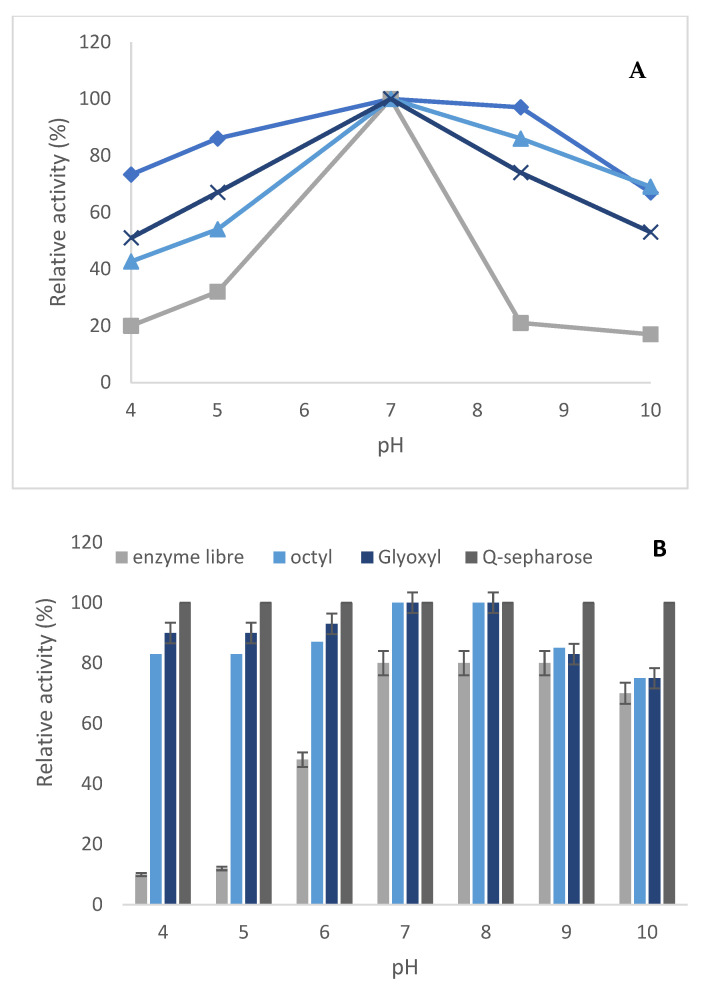
Effect of pH on the activity (**A**) and stability (**B**) of PLC_Bc_ soluble and immobilized on octyl sepharose, Q-sepharose, and glyoxyl agarose in the presence of N-acetyl cysteine. (**A**) Blue squares: Q-sepharose, blue cross: glyoxyl agarose, blue triangles: octyl sepharose, gray: soluble enzyme.

In order to check if the modification in the reaction medium may affect the enzyme activity of the soluble and immobilized enzymes, the activity of both free enzymes and three immobilized derivatives on pNPPC hydrolysis was determined in the pH range between 4 and 10, and the results are presented in Figure 4. The optimum pH of the free and immobilized enzymes on octyl, glyoxyl agarose, and Q-sepharose was similar. Furthermore, the optimum activity was determined with phosphate buffer at pH 7. The retained activity of the immobilized enzyme on the three supports was enhanced at lower and higher pHs in comparison to the free enzyme. Moreover, the three derivatives presented a flat profile in all the ranges of considered conditions. These results show that immobilization increases its activity at different pHs with respect to the soluble enzyme. It is an advantage, since it allows for the extension of the pH range in the working conditions of different reactions.

The stability against various pH values ranging between 4 and 10 at 37 °C during 4 h of incubation of the free and the three immobilized PLC_Bc_ was compared. At different pH conditions, the various derivatives of PLC_Bc_ were more stable than the soluble enzyme (Figure 4). At acid pH (4 and 5), the soluble enzyme lost 35% of its residual activity, whereas the enzyme immobilized on Q-sepharose and glyoxyl agarose lost, respectively, 20 and 15% of their residual activity. On the other hand, the enzyme immobilized on octyl maintains all its residual activity at different pH conditions. In addition, the obtained results could be attributed to the capacity of the micro-environment generated between the support and the soluble enzyme, permitting the protection of the latter from denaturation that can be caused by a change in pH conditions and also a change in the buffer’s nature and in the ionic strength. Nevertheless, the immobilization technique has a great effect on the stabilization of the enzyme.

### 2.3. Thermal Stability of PLC_Bc_ Immobilized on Different Supports

The thermal stability of the soluble and the various immobilized enzymes was determined after their incubation at various temperatures (50 °C, 60 °C, and 70 °C at pH 7) in 10 mM sodium phosphate buffer for different periods of time. 

**Figure 5:**
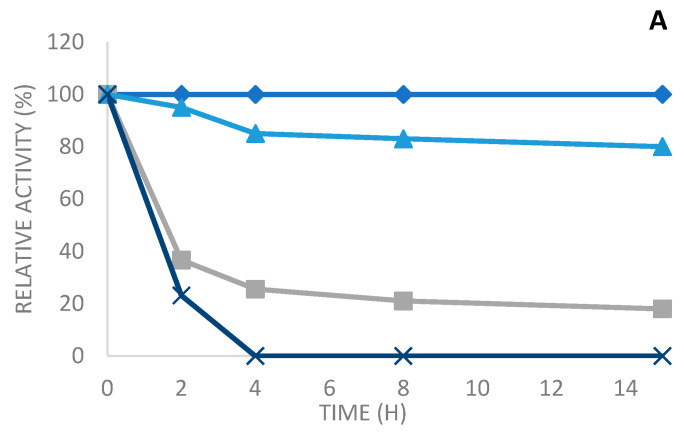

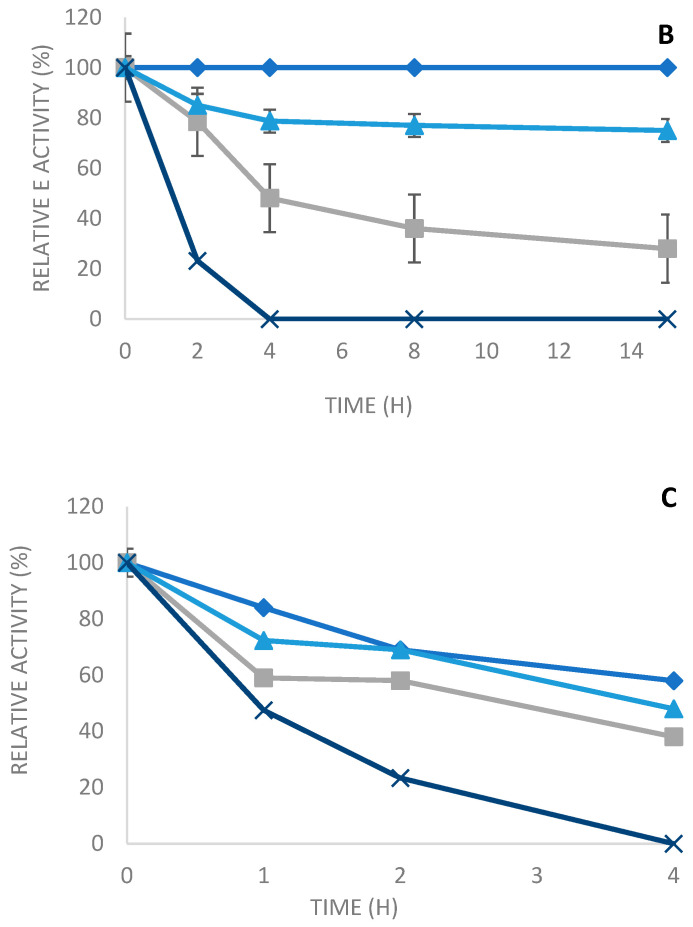
Thermal inactivation at pH 7, 50 °C (**A**), 60 °C (**B**), and 70 °C (**C**) of soluble enzymes and various derivatives: octyl sepharose, Q-sepharose, and glyoxyl agarose in the presence of N-acetylcysteine. Blue squares: octyl sepharose, blue triangles: glyoxyl sepharose, gray: Q-sepharose, and blue cross: soluble enzyme.

As seen in Figure 5, the three derivatives are very stable compared to the soluble enzyme, which is also a thermostable enzyme. Then, the soluble enzyme is almost entirely inactivated in the course of the inactivation time at all the tested temperatures. On the other side, the different immobilized enzymes maintained the activity over time. 

Thus, at 50 °C, PLC_Bc_ immobilized on octyl sepharose and glyoxyl agarose in the presence of N-acetyl cysteine were highly stable; they maintained, respectively, 100% and 80% of residual activity after 15 h of incubation (Figure 5A). However, the Q-sepharose derivative was less stable when compared to the two other derivatives, reaching a residual activity of 25% after 4 h of incubation at 50 °C and an inactivation of 82% after 15 h (Figure 5A). On the other hand, the soluble enzyme was totally inactivated after 4 h of incubation at 50 °C (Figure 5A). At 60 °C, as shown in Figure 5B, after heat treatment for 15 h and pH 7, the full initial activity was maintained with the enzyme immobilized on octyl sepharose. Furthermore, a more than a 75% drop in the initial activity was observed in the immobilized enzyme on glyoxyl agarose in the presence of N-acetyl cysteine, whereas it attained only 28% for the immobilized enzyme on Q-sepharose. Nevertheless, the free enzyme was completely inactivated after 4 h of incubation at 60 °C. This result clearly displays the efficiency of the immobilization method to protect enzymes against heat inactivation. Other studies [34,35] confirm the improved thermal stability of the immobilized enzyme compared to the soluble enzyme. 

The results obtained from the experiments performed at 70 °C (Figure 5) show that the highest stability was acquired with PLC_Bc_ immobilized on octyl sepharose. Therefore, there was a reduction in residual activity of just 52%, which was 50-fold more stable than the free enzyme. After 4 h of incubation, the derivative of octyl sepharose was 50-fold more stable than the free enzyme, with a reduction in its residual activity of only 52%. The PLC_BC_ immobilized on glyoxyl agarose in the presence of N-acetyl cysteine also appeared to have good stability (48-fold more stable than the soluble enzyme), reaching a residual activity of 48% after 4 h of incubation at 70 °C. On the other hand, Q-sepharose had a residual activity of only 38-fold more than the free enzyme. 

When we compare the thermal stability to other derivatives of the soluble enzyme and the different immobilization techniques of the PLC_Bc_, it can be observed that the obtained results are quite similar, as seen in the profiles of the various derivates at the three tested temperatures. The derivatives immobilized on octyl sepharose are more stable than, than, respectively, the derivatives immobilized by glyoxyl agarose in the presence of N-acetyl cysteine and afterward on Q-sepharose. 

### 2.4. Effect of Organic Solvents on Soluble Enzymes and Derivatives

In some biological applications, the reaction catalyzed by phospholipase could be realized in the presence of organic solvents. Nevertheless, it was common that enzyme activity could be affected by the presence of organic solvents, which could provide a waste of the catalytic activity through the denaturation of the enzyme.

As a matter of fact, the phospholipase activity of the soluble enzyme and the derivatives was determined in the presence of various organic solvents; the effect of various solvents (methanol, ethanol, acetone, and hexane) was checked in the presence of 50% of the solvents for 4 h at room temperature.

As shown in Table 2, the enzyme activity of most of the preparations maintained very high levels during the inactivation times (after 2 h and 4 h). Also, some preparations showed an increase in enzyme activity in the presence of some organic solvents like ethanol and hexane. This hyperactivation could be explained by some conformational modifications caused by the enzyme under the initial incubation conditions. The enzymes with the highest increase in activity were immobilized on octyl sepharose and glyoxyl agarose in the presence of N-acetyl cysteine.

The enzyme with the highest enzyme activity was immobilized on octyl sepharose with ethanol (293.93%). This derivative also has a high hyperactivation in hexane (251.06%). With the same solvent, the highest increase in enzyme activity was found with the derivative of glyoxyl agarose in the presence of N-acetylcysteine, with a residual activity of 289.78%. The other immobilized preparation on Q-sepharose was also highly stable in all the tested organic solvents. It maintained around 85% of its residual activity in the presence of acetone and its full activity (100%) in the presence of 50% (*v*/*v*) of methanol. Moreover, the derivative of Q-sepharose also showed hyperactivation in the presence of ethanol and hexane, with a residual activity of 234.36% and 185.91%, respectively.

### 2.5. Effect of Storage on the Soluble and the Immobilized Enzyme

In order to study the long-term storage stability, the different enzyme preparations (soluble and the various derivatives) were stored at 4 °C.

**Figure 6:**
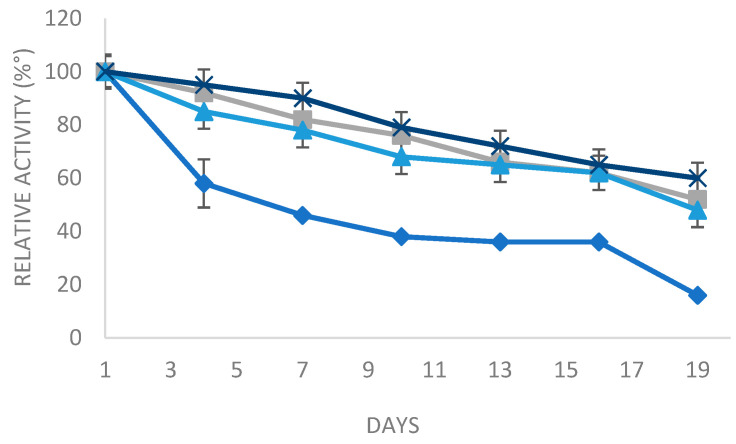
Residual activity of the soluble enzyme and the three derivatives as affected by incubation at 4 °C from 1 to 19 days. Bars indicate standard deviation. Blue squares: soluble enzyme, blue triangles: Q-sepharose, gray: octyl sepharose, and blue cross: glyoxyl agarose.

As represented in Figure 6, it could be observed that the storage stability of the immobilized enzyme on different supports was clearly higher than the free enzyme. In fact, after a storage period of 19 days, the derivative glyoxyl agarose in the presence of N-acetyl cysteine, octyl sepharose, and Q-sepharose could still maintain, respectively, above 60%, 52%, and 48% of the residual activity. The derivative of glyoxyl agarose in the presence of N-acetylcysteine has lower storage stability (40% left over 19 days). Nevertheless, the soluble enzyme preserves only 20% of its initial activity. In conclusion, the immobilization of PLC_Bc_ is a sustainable method in terms of maintaining the long-term storage stability of the enzyme.

## 3. Materials and Methods 

### 3.1. Reagents

*p*-Nitrophenylphosphorylcholin (pNPPC) was purchased from Sigma-Aldrich (Sigma-Aldrich, Darmstadt, Germany), it was used as substrate for the phospholipase C assay, tryptone, yeast extract, NaCl, Q-sepharose, glyoxyl, Tris-Hcl, Zncl_2_, sodium phosphate, bicarbonate buffer, N-acetyl cysteine, sodium borohydride, SDS, and polyacrylamide were purchased from Sigma Chemical Germany, also B-mercaptoethanol, blue de coomassie, sodium acetate, sodium bicarbonate, methanol, ethanol, acetone, isopropanol, and hexane. All reagents and solvents were of analytical grade.

### 3.2. Microorganism and Enzyme Production

The current study reports on a novel phospholipase C from *Bacillus cereus* (PLC_Bc_) isolated from oily soil (olive oil and soy lecithin) located at Sfax-Tunisia [28]. The sequence of the 16S rRNA gene was deposed in the GenBank database, and it can be identified by the access number OR801792. 

The PLC_Bc_ was produced under optimized conditions, as already described [28]. Phospholipase C was produced by immersed fermentation. The bacterial strain was pre-cultured in a medium with the following composition: 5 g/L yeast extract, 10 g/L tryptone, 10 g/L NaCl, and pH 7.5. The pre-culture was incubated for 15 h at 200 rpm and 37 °C in 250 mL culture flasks. 

The pre-culture was inoculated with a medium containing 10 g/L yeast extract, 10 g/L tryptone, 8.125 g/L NaCl, 0.15 as the initial OD at 600 nm, and pH 7.5. The culture was released at a temperature of 37 °C and 200 rpm on a rotary shaker. 

At the end of cultivation (6 h), the enzymatic crude of phospholipase C was acquired after centrifugation at 8500 g for 10 min in order to remove the bacterial cells. The supernatant obtained from the culture medium containing extracellular phospholipase was used as the crude enzyme. The latter was precipitated with ammonium sulfate with a saturation range of 80% under gentle stirring at 4 °C for 1 h. After centrifugation for 20 min at 12,000 rpm, the obtained product was dissolved in a buffer containing 150 mM phosphate, 150 mM NaCl, pH 7.2, and subjected to dialysis at 4 °C in order to remove residual ammonium sulfate. The obtained enzyme solution was then applied to the immobilization process. 

### 3.3. Phospholipase C Assay 

The phospholipase C activity of the soluble enzyme and the derivatives was determined spectrophotometrically by the hydrolysis of pNPPC. PLC_Bc_ activity was tested out according to the modified protocol of [36]. The assays were released at 405 nm by measuring the increase in nitrophenol produced by the hydrolysis of pNPPC at 405 nm. The reaction solution contained 60 microliters of the PLC enzyme solution, which was added to 2 mL of pNPPC solution, 250 mmol/L Tris-HCl (pH 7.2), 60% sorbitol, and 1 mmol/L ZnCl_2_. One international unit of activity (U) was elucidated as the quantity of enzyme that hydrolyzed 1 μmol of pNPPC per minute under standard conditions, as described previously. 

The suspension containing the derivatives was gutted by a pipette-tip filter, and its activities were released by cut-pipette-tips.

### 3.4. Immobilization of PLC_Bc_ on Octyl Support

Immobilization of PLC_Bc_ was carried out using 5 mg of protein per g of support. The enzyme was diluted in 25 mM sodium phosphate buffer, pH 7, under stirring at 210 rpm at room temperature. The yield of immobilization was checked at various times. Moreover, the enzyme activity was checked in both the supernatant and suspension solutions at regular time intervals. The immobilization reaction was finalized when the activity in the supernatant was zero. The immobilization enzyme biocatalyst was recovered by filtration and was washed several times with distilled water.

The derivate was stored at 4 °C until use. The recovery activity (%) was calculated as follows:

100 × (a_0_ − a)/a_0_ (1)

where a_0_ is the total enzyme activity and is the number of units added to the supports for the immobilization reaction (the total initial activity).

a is the enzyme activity after immobilization.

### 3.5. Immobilization of PLC_Bc_ on Q-Sepharose Support

Immobilization of PLC_Bc_ was performed using 5 mg of enzyme per g of Q-sepharose support. The enzyme was diluted in 25 mM sodium phosphate buffer, pH 7. The immobilization process was carried out at room temperature under stirring at 210 rpm. The yield of immobilization was checked periodically in the supernatant and the suspension. When the immobilization was finished, the derivate was filtrated, washed with distilled water, and dried by suction. Finally, the immobilization preparations were stored at 4 °C.

### 3.6. Immobilization of PLC_Bc_ on Glyoxyl Agarose in the Presence of N-Acetyl Cysteine Support

A total of 5 mg of enzyme per g of glyoxyl agarose support was immobilized in 25 mM bicarbonate buffer and in the presence of 50 mM N-acetylcysteine. The immobilization process was carried out at room temperature and under stirring at 210 rpm. At the end of immobilization, a ratio of 1 mg/mL of sodium borohydride was added to the reaction mixture under vigorous stirring for 30 min at room temperature in order to reduce the base’s de Schiff bonds and have irreversible covalent attachment.

Then, the derivate was treated in the same way as explained in Section 3.4. The yield and recovered activity after the immobilization reaction were measured as reported in the previous immobilization process.

### 3.7. S-PAGE Analysis of Enzyme Immobilization

SDS-polyacrylamide gel electrophoresis 12% was released following the protocol of [37]. Samples containing the same amount of protein were taken from different enzyme preparations. Then, these alliquots were re-suspended and boiled in the presence of SDS and β-mercaptoethanol in order to dissolve the proteins not covalently attached to the support [38]. After migration, the gels were stained by coomassie blue staining to detect the proteins, according to the literature.

### 3.8. Effect of pH on Free and Immobilized Enzyme Activity and Stability

The effect of pH on the soluble enzyme and different derivatives was pursued by constantly determining the enzyme activity as described in material and methods Section 3.3, over the pH range of 4–10 using the following buffers at 50 mM: sodium acetate pH 4–6, Tris-HCL pH 7, and sodium bicarbonate pH 8–10.

Then, in order to study the pH stability, the free enzyme and different derivates were incubated to stand for 4 h at various values (pH 4 to 10) at 37 °C, and then the residual activities were determined.

### 3.9. Thermal Inactivation of Free and Immobilized Enzymes

The thermal stability of the different derivates was determined by suspending 0.2 g of each biocatalyst in 2 mL of sodium phosphate buffer, pH 7. Then, the various preparations were incubated at 50 °C, 60 °C, and 70 °C. Periodically, aliquots from each sample were withdrawn, and the enzymatic activity was determined as described above. Residual activity was mentioned as the ratio between the activity at a given time and the initial activity.

### 3.10. Effect of Organic Solvents on Soluble and Immobilized Enzymes

Samples of the different enzyme preparations (soluble enzyme and different immobilized enzyme) were incubated in the presence of 50% solvents (methanol, ethanol, acetone, and hexane). Periodically, aliquots from each sample were withdrawn, and the enzyme’s activity was measured as described above.

### 3.11. Enzyme Storage Stability

Samples of soluble and immobilized enzymes were reserved at 4 °C for 19 days, and the residual activity was determined every 3 days.

### 3.12. Statical Analysis

All experiments were determined in triplicate, and the results are expressed as the mean ± SE.

## 4. Conclusions

In the present study, a novel phospholipase C from *Bacillus cereus* isolated from oily soil located in Sfax, Tunisia was immobilized using various immobilization methods. Different supports were checked: octyl sepharose and glyoxyl agarose in the presence of N-acetyl cysteine and Q-sepharose. It was determined that the PLC_Bc_ was immobilized on different supports with the high recovery activity of 80% and 58% after immobilization on Q-sepharose and glyoxyl agarose in the presence of N-acetyl cysteine, respectively. A 2-fold hyperactivation was obtained during immobilization by hydrophobic support using octyl sepharose.

The biochemical characterization of the immobilized PLC_Bc_ was compared with that of the soluble enzyme. In conclusion, all the immobilized enzymes were more stable than the soluble enzyme. The derivatives acquired using octyl sepharose, glyoxyl agarose, and Q-Sepharose were, respectively, stable at high temperatures (50 °C, 60 °C, and 70 °C). Nevertheless, the three derivatives were stable in a wider range of pHs than the soluble enzyme. The three derivatives and the free enzyme were stable in 50% (*v*/*v*) ethanol, hexane, methanol, and acetone. The glyoxyl agarose derivative showed high long-term storage at 4 °C, with an activity of 60% after 19 days. In fact, what the most stable derivative is depends on the inactivation conditions.

In this study, the obtained biochemical properties manifested the potential of immobilization for the improvement of the biochemical characteristics of the enzyme without real applicability. The sustainable biotechnological application of the various immobilized enzymes could be carried out in the future under specific reaction conditions. This matter requires further research by our group.

## Figures and Tables

**Table 1:** Recovery activity after immobilization and hyperactivation factor during immobilization of PLC_Bc_ on various supports.

Support	Recovery Activity after Immobilization	Hyperactivation Factor (Fold)
Octyl	192.5	1.92
DEAE	85	-
Glyoxyl agarose	58.18	-

**Table 2:** Residual activity of the soluble enzyme and the three derivatives as affected by incubation for 4 h at 37 °C in the presence of 50% of organic solvents (hexane, acetone, ethanol, and methanol). Aliquots were taken at 2 h and 4 h.

Solvents	Time (h)	Soluble Enzyme	Octyl	Q-Sepharose	Glyoxyl Agarose
**Acetone**	2	76.35	100	81	100
	4	41.51	87.7	55	63.97
**Ethanol**	2	100	293.93	237.36	100
	4	82.90	105.92	100	100
**Methanol**	2	100	100	100	100
	4	100	100	100	100
**Hexane**	2	72.23	272.93	95.48	289.78
	4	38.68	251.06	85.91	223.66

## Data Availability

Materials, data, and associated protocols are available to readers without undue qualifications regarding material transfer agreements. For data retrieval, please contact (email: g.f.lorente@csic.es).
